# 14-3-3γ Regulates Lipopolysaccharide-Induced Inflammatory Responses and Lactation in Dairy Cow Mammary Epithelial Cells by Inhibiting NF-κB and MAPKs and Up-Regulating mTOR Signaling

**DOI:** 10.3390/ijms160716622

**Published:** 2015-07-22

**Authors:** Lixin Liu, Ye Lin, Lili Liu, Yanjie Bian, Li Zhang, Xuejun Gao, Qingzhang Li

**Affiliations:** Key Laboratory of Dairy Science of Education Ministry, Northeast Agricultural University, Harbin 150030, China; E-Mails: liulixin_2003@163.com (Lix.L.); linlu516@163.com (Y.L.); liulili-liulili@163.com (Lil.L.); bianjie.033@163.com (Y.B.); zhangli_zl18800@163.com (L.Z.); gxjgxj112233@163.com (X.G.)

**Keywords:** 14-3-3γ, inflammatory responses, dairy cow mammary epithelial cells, NF-κB, MAPKs, mTOR signaling pathway

## Abstract

As a protective factor for lipopolysaccharide (LPS)-induced injury, 14-3-3γ has been the subject of recent research. Nevertheless, whether 14-3-3γ can regulate lactation in dairy cow mammary epithelial cells (DCMECs) induced by LPS remains unknown. Here, the anti-inflammatory effect and lactation regulating ability of 14-3-3γ in LPS-induced DCMECs are investigated for the first time, and the molecular mechanisms responsible for their effects are explored. The results of qRT-PCR showed that 14-3-3γ overexpression significantly inhibited the mRNA expression of tumor necrosis factor-α (TNF-α), interleukin-6 (IL-6), interleukin-1β (IL-1β) and inducible nitric oxide synthase (iNOS). Enzyme-linked immunosorbent assay (ELISA) analysis revealed that 14-3-3γ overexpression also suppressed the production of TNF-α and IL-6 in cell culture supernatants. Meanwhile, CASY-TT Analyser System showed that 14-3-3γ overexpression clearly increased the viability and proliferation of cells. The results of kit methods and western blot analysis showed that 14-3-3γ overexpression promoted the secretion of triglycerides and lactose and the synthesis of β-casein. Furthermore, the expression of genes relevant to nuclear factor-κB (NF-κB) and mitogen-activated protein kinase (MAPKs) and lactation-associated proteins were assessed by western blot, and the results suggested that 14-3-3γ overexpression inactivated the NF-κB and MAPK signaling pathways by down-regulating extracellular signal regulated protein kinase (ERK), p38 mitogen-activated protein kinase (p38MAPK) and inhibitor of NF-κB (IκB) phosphorylation levels, as well as by inhibiting NF-κB translocation. Meanwhile, 14-3-3γ overexpression enhanced the expression levels of β-casein, mammalian target of rapamycin (mTOR), ribosomal protein S6 kinase 1 (S6K1), serine/threonine protein kinase Akt 1 (AKT1), sterol regulatory element binding protein 1 (SREBP1) and peroxisome proliferator-activated receptor gamma (PPARγ). These results suggest that 14-3-3γ was able to attenuate the LPS-induced inflammatory responses and promote proliferation and lactation in LPS-induced DCMECs by inhibiting the activation of the NF-κB and MAPK signaling pathways and up-regulating mTOR signaling pathways to protect against LPS-induced injury.

## 1. Introduction

As a common metabolic disease, the incidence of sub-acute ruminal acidosis (SARA) is greater than 20% in the primary and middle lactation of dairy cows, which causes significant economic damage to dairy cattle cultivation [1]. When SARA occurs, the bacterial lipopolysaccharide (LPS) concentrations are significantly increased [2]. LPS, as a major component of the outer membrane of Gram-negative bacteria, is usually viewed as a highly efficient proinflammatory response factor that activates the nuclear factor-κB (NF-κB) and mitogen-activated protein kinase (MAPK) pathways and ultimately results in the release of a large number of pro-inflammatory cytokines, such as tumor necrosis factor-α (TNF-α), interleukin-6 (IL-6), IL-1β and so on [3,4,5,6]. Many studies have shown that proinflammatory cytokines in dairy cows can lead to chronic mastitis [7], changes in nutritional metabolism and cells apoptosis which can ultimately influence the milk composition and milk yield [8,9,10,11]. Therefore, it remains a formidable challenge to inhibit the inflammatory cytokine production induced by LPS, thereby improving the cow’s milk quality. Previous studies have shown that LPS initiates the inflammatory response, and at the same time, the body has a complex negative regulatory network to down-regulate the inflammatory response; thus, the inflammatory response induced by LPS is regulated by the body. Recently, several intracellular signaling-regulated molecules have been identified [12,13]. However, in contrast to many well-studied signaling molecules, 14-3-3γ remains relatively uncharacterized.

The 14-3-3 proteins family are highly conserved dimeric proteins, these have serine/threonine phosphorylation binding sites in mammals and have seven subunits (β, γ, ε, ζ, η, σ and τ). The family members play particularly important roles in cell biology due to their involvement in vital cellular processes, such as metabolism, protein trafficking, signal transduction, apoptosis and cell-cycle regulation [14]. 14-3-3γ is an influential member of the 14-3-3 family, with subcellular localization in the cell nucleus [15]. In most cells, 14-3-3γ is critical for maintaining cellular homeostasis and signal transduction [16]. Moreover, 14-3-3γ is also an important factor for intracellular phosphorylation, which participates in various pathophysiological processes [17]. Our prior research has shown that 14-3-3γ overexpression can promote the proliferation of dairy cow mammary epithelial cells (DCMECs) and increase cell viability [18]. Notably, in our ongoing research, we recently also confirmed that 14-3-3γ can promote the protein expression of mammalian target of rapamycin (mTOR) and serine/threonine protein kinase-1 (AKT1), and thereby promote the secretion of milk protein and milk fat and improve DCMEC lactation ability. More recently, 14-3-3γ was shown to protect against LPS-induced cell injury, likely through a pathway associated with prevention of mitochondrial permeability transition pore (mPTP) opening, and ultimately the inhibition of apoptosis [19,20]. However, it remains unclear whether 14-3-3γ is able to have a protective effect on LPS-induced DCMECs.

Thus, the aim of the current study was designed to investigate the anti-injury effects and mechanisms of 14-3-3γ in LPS-induced DCMECs. Herein, we have examined the expression of pro-inflammatory cytokines and the activity of the MAPK and NF-κB signaling pathways. In addition, the secretion of β-casein, triglycerides, and lactose, as well as lactation related transcription factors (mTOR, ribosomal protein S6 kinase (S6K1), serine/threonine protein kinase Akt (AKT), sterol regulatory element binding protein 1 (SREBP1), peroxisome proliferator-activated receptor gamma (PPARγ), and Glucose transporter 1 (GLUT1)), have been evaluated.

## 2. Results

### 2.1. Identification of Dairy Cow Mammary Epithelial Cells (DCMECs)

**Figure 1:**
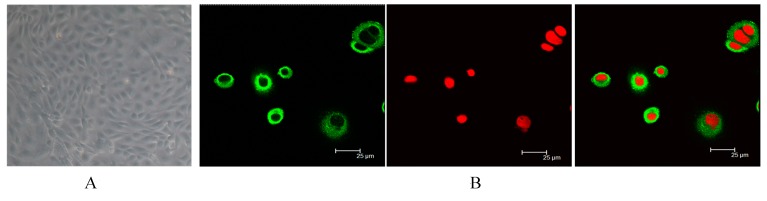
Morphology of cultured and identified dairy cow mammary epithelial cells (DCMECs). (**A**) Purified epithelial cells were obtained by digestion (400×); (**B**) Purified DCMECs expressed cell marker cytokeratin 18 (CK18). CK18 was counterstained with fluorescein isothiocyanate (FITC), and nuclei were counterstained with propidium iodide (PI).

The morphology and cell marker cytokeratin 18 (CK18) expression of the isolated and cultured cells is shown in Figure 1. The purified cells were (Figure 1A) and were identified by detecting the expression of CK18. Positive staining for CK-18 was shown in bovine epithelial cells (Figure 1B). Overall, the staining indicates that DCMECs were successfully cultured in this study.

### 2.2. Effect of Lipopolysaccharide (LPS) on the Expression of 14-3-3γ mRNA

**Figure 2:**
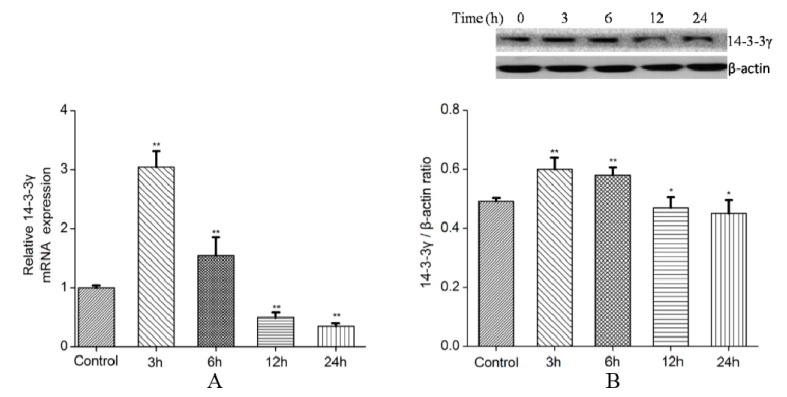
Effect of lipopolysaccharide (LPS) on the expression of 14-3-3γ in DCMECs. DCMECs were treated with 1 μg/mL LPS for 0, 3, 6, 12 and 24 h, relative expression levels of 14-3-3γ were determined by qRT-PCR and western blot (**A**) The relative expression of 14-3-3γ mRNA; (**B**) The relative expression of 14-3-3γ protein. All of these expressions were normalized to a housekeeping gene (*β-actin*). Bars indicate the mean ± SD (*n* = 3). * *p* < 0.05 and ** *p* < 0.01 *vs.* control group.

The mRNA expression of 14-3-3γ was changed when DCMECs were stimulated with LPS. DCMECs were treated with 1 μg/mL LPS for 0, 3, 6, 12 and 24 h, and the expression level of 14-3-3γ mRNA increased significantly at 3–6 h compared to the control group (untreated cells were used as a control) (*p* < 0.01), then its expression level declined at 12–24 h compared to the control group (*p* < 0.01) (Figure 2).

### 2.3. Expression of 14-3-3γ Proteins

**Figure 3:**
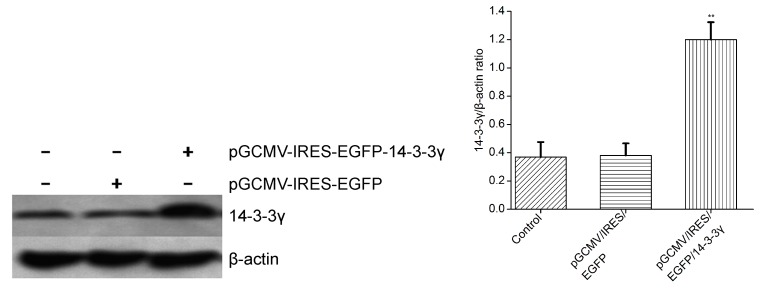
The protein expression of 14-3-3γ in DCMECs that were transfected with pGCMV-IRES-EGFP-14-3-3γ (expression vectors) for 24 h. The relative expression of the 14-3-3γ protein was determined by normalization to *β-actin*. Bars indicate the mean ± SD (*n* = 3). ** *p* < 0.01 *vs.* control group.

DCMECs were transfected with pGCMV-IRES-EGFP-14-3-3γ (expression vectors) or pGCMV-IRES-EGFP for 24 h. The total proteins were extracted and analyzed by western blot for 14-3-3γ. The results show that the relative expression of 14-3-3γ protein in DCMECs transfected with pGCMV-IRES-EGFP-14-3-3γ increased significantly compared to the control group (the absence of pGCMV-IRES-EGFP-14-3-3γ) (*p* < 0.01) (Figure 3). These results demonstrate that the pGCMV-IRES-EGFP-14-3-3γ expression vector was effectively expressed in cultured DCMECs.

### 2.4. 14-3-3γ Overexpression Inhibited Inflammatory Cytokine mRNA Expression in LPS-Induced DCMECs

**Figure 4:**
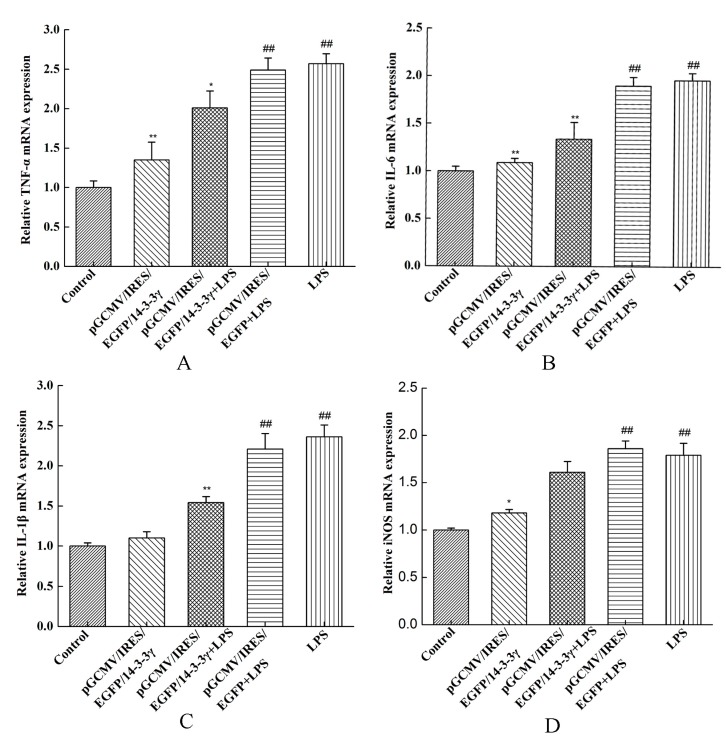
Effects of 14-3-3γ overexpression on cytokine mRNA expression in LPS-induced DCMECs. DCMECs were pre-transfected with pGCMV-IRES-EGFP-14-3-3γ or pGCMV-IRES-EGFP. After 24 h, the cells were stimulated with LPS (1 μg/mL) for 6 h. The mRNA expression of tumor necrosis factor-α (TNF-α), interleukin-6 (IL-6), interleukin-1β (IL-1β) and inducible nitric oxide synthase (iNOS) were measured by RT-PCR. (**A**) TNF-α expression; (**B**) IL-6 expression; (**C**) IL-1β expression; (**D**) iNOS expression. All of these expressions were normalized to a housekeeping gene (*β-actin*). Bars indicate the mean ± SD (*n* = 3). * *p* < 0.05 and ** *p* < 0.01 *vs.* LPS group; ^##^ *p* < 0.01 *vs.* control group.

The mRNA expression of TNF-α, IL-6, IL-1β and inducible nitric oxide synthase (iNOS) were measured by qRT-PCR. The results showed that treatment with LPS alone resulted in a significant increase in cytokine expression compared to the control group (*p* < 0.01). However, 14-3-3γ overexpression significantly decreased the LPS-induced expression of TNF-α, IL-6 and IL-1β compared to the LPS group (*p* < 0.05 or *p* < 0.01), but iNOS had little change (Figure 4).

### 2.5. 14-3-3γ Overexpression Decreased the Production of Inflammatory Cytokines in LPS-Induced DCMECs

**Figure 5:**
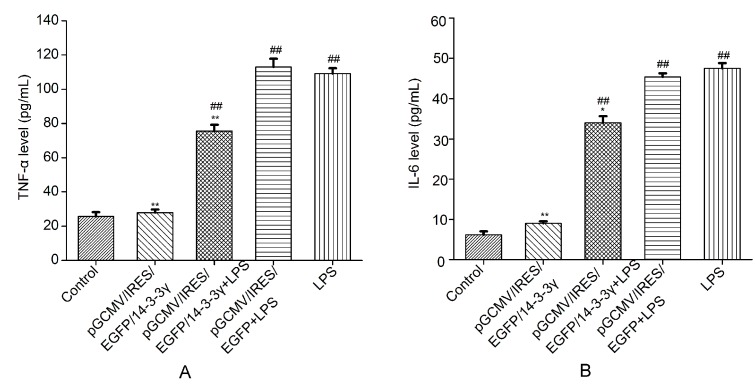
Effect of 14-3-3γ overexpression on the production of inflammatory cytokines in LPS-induced DCMECs. DCMECs were pre-transfected with pGCMV-IRES-EGFP-14-3-3γ or pGCMV-IRES-EGFP. After 24 h, the cells were stimulated with LPS (1 μg/mL) for 6 h. The levels of TNF-α and IL-6 were detected by Enzyme-linked immunosorbent assay (ELISA). (**A**) TNF-α level; (**B**) IL-6 level. Bars indicate the mean ± SD (*n* = 3). * *p* < 0.05 and ** *p* < 0.01 *vs.* LPS group; ^##^ *p* < 0.01 *vs.* control group.

LPS-induced inflammation develops due to the secretion of various pro-inflammatory mediators. To determine whether 14-3-3γ overexpression affects the production of inflammatory cytokines in LPS-induced DCMECs, the levels of TNF-α and IL-6 were detected by ELISA. Our results demonstrated that treatment of DCMECs with LPS alone resulted in a remarkable increase in TNF-α and IL-6 production compared to control group (*p* < 0.01). However, the 14-3-3γ overexpression plasmid (pGCMV-IRES-EGFP-14-3-3γ) decreased the LPS-induced production of TNF-α and IL-6 in the cell culture supernatants compared to the LPS group (*p* < 0.01) (Figure 5).

### 2.6. 14-3-3γ Overexpression Inhibited the Mitogen-Activated Protein Kinase (MAPK) Signal Pathway in LPS-Induced DCMECs

**Figure 6:**
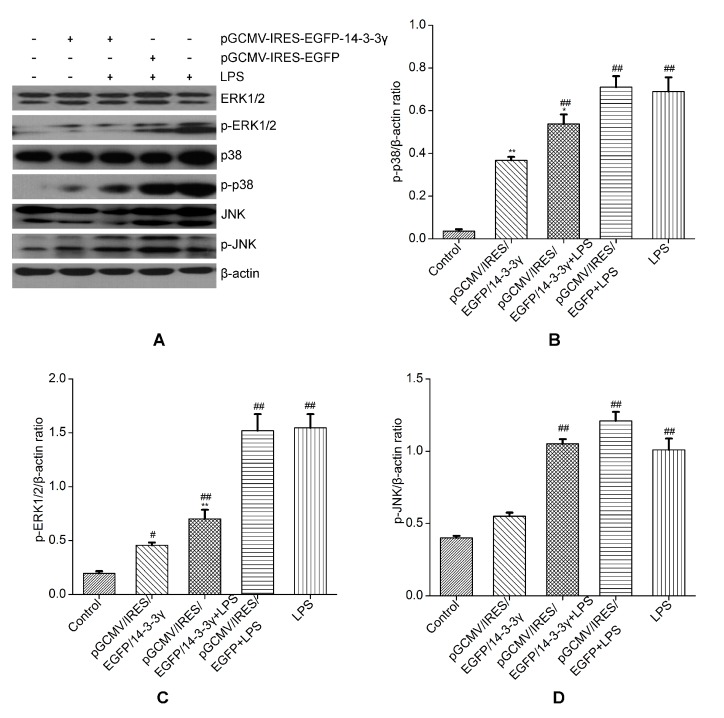
Effects of 14-3-3γ overexpression on the mitogen-activated protein kinase (MAPK) signaling pathway in LPS-induced DCMECs. DCMECs were pre-transfected with pGCMV-IRES-EGFP-14-3-3γ or pGCMV-IRES-EGFP. After 24 h, the cells were stimulated with LPS (1 μg/mL) for 1 h. Cytosolic proteins were analyzed by western blot for extracellular signal regulated protein kinase 1/2 (ERK 1/2), p38 mitogen-activated protein kinase (p38 MAPK) and Junamino terminal kinase (JNK) phosphorylation. (**A**) Western blot detection of mentioned proteins; (**B**) Quantitation of p-p38; (**C**) Quantitation of p-ERK1/2; (**D**) Quantitation of p-JNK. *β-Actin* was used as a control. Bars indicate the mean ± SD (*n* = 3). * *p* < 0.05 and ** *p* < 0.01 *vs.* LPS group; ^#^ *p* < 0.05 and ^##^ *p* < 0.01 *vs.* control group.

MAPKs, including extracellular signal regulated protein kinase (ERK), p38 mitogen-activated protein kinase (p38 MAPK) and Junamino terminal kinase (JNK), are critical signaling molecules in the cell signaling processes mediated by LPS. The MAPK pathway takes part in the regulation of TNF-α release in LPS-induced cells [21]. To confirm whether the inhibition of the inflammatory response by 14-3-3γ overexpression is mediated through the MAPK pathway, phosphorylation of JNK, ERK and p38 MAPKs were examined by western blot. The results showed that LPS-induced cells had significantly increased phosphorylation of ERK1/2 and p38 MAPK, JNK, compared to the control group (*p* < 0.01). However, 14-3-3γ overexpression inhibited the increase in phosphorylation of ERK1/2 and p38 MAPK in LPS-induced DCMECs compared to the LPS group (*p* < 0.05 or *p* < 0.01), but without inhibitory effect on phosphorylation of JNK (Figure 6).

### 2.7. 14-3-3γ Overexpression Inhibited the Nuclear Factor-κB (NF-κB) Signaling Pathway in LPS-Induced DCMECs

**Figure 7:**
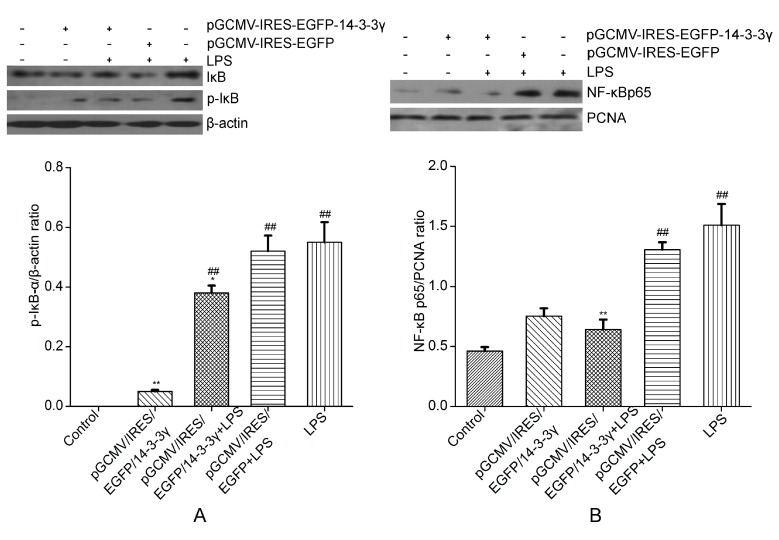
Effects of 14-3-3γ overexpression on the nuclear factor-κB (NF-κB) signaling pathway in LPS-induced DCMECs. DCMECs were pre-transfected with pGCMV-IRES-EGFP-14-3-3γ or pGCMV-IRES-EGFP. After 24 h, the cells were stimulated with LPS (1 μg/mL) for 1 h. (**A**) Cytosolic proteins were analyzed by western blot for phosphorylated inhibitor inhibitor of NF-κB (IκB). *β-Actin* was used as a control; (**B**) Nuclear proteins were analyzed by western blot for nuclear factor-κB p65 (NF-κB p65). Proliferating cell nuclear antigen (PCNA) was used as a control. Bars indicate the mean ± SD (*n* = 3). * *p* < 0.05 and ** *p* < 0.01 *vs.* LPS group; ^##^ *p* < 0.01 *vs.* the control group.

In resting cells, NF-κB is present in the cytosol in an inactive state complexed with its inhibitor inhibitor of NF-κB (IκB) proteins [22,23]. When the cells are stimulated by LPS, IκB is phosphorylated and nuclear translocation of active NF-κB occurs. To confirm whether the inhibition of the inflammatory response by 14-3-3γ is mediated through the NF-κB pathway, the degree of phosphorylation of IκB was measured by western blot. The results showed that LPS-induced cells had significantly increased phosphorylation of IκB compared to the control group (*p* < 0.01). However, 14-3-3γ inhibited the increase of IκB phosphorylation in LPS-induced cells compared to the LPS group (*p* < 0.01) (Figure 7A).

To test the expression of NF-κB proteins in the nucleus, the nuclear proteins were extracted and evaluated for NF-κB translocation. The results show that NF-κB translocation was detected in the nuclear proteins of LPS-induced cells. However, 14-3-3γ significantly inhibited LPS-induced NF-κB translocation (*p* < 0.01) (Figure 7B).

### 2.8. 14-3-3γ Overexpression Improved the Viability and Proliferation of LPS-Induced DCMECs

To investigate whether cell viability and proliferation is related to decreased cytokine production, DCMECs were pre-transfected with pGCMV-IRES-EGFP-14-3-3γ or pGCMV-IRES-EGFP for 24 h and then were stimulated with LPS (1 μg/mL). After 24 h, cell viability and proliferation were determined using the CASY-TT Analyzer System (Schärfe System GmbH, Reutlingen, Germany). The results showed that LPS inhibited the viability and proliferation of cells compared to the control group (*p* < 0.01). However, 14-3-3γ overexpression could not only increase the viability and proliferation of normal DCMECs compared to the control group (*p* < 0.05) but also clearly increased the viability and proliferation of LPS-induced DCMECs compared to the LPS group (*p* < 0.01) (Table 1).

**Table 1:** Viability and proliferation of 14-3-3γ overexpression on LPS-induced DCMECs.

Groups	Numbers (10^5^)	Viability (%)
Control	5.15 ± 0.21	95.51 ± 5.36
pGCMV-IRES-EGFP-14-3-3	5.97 ± 0.37 *	97.08 ± 4.99 *
pGCMV-IRES-EGFP	5.39 ± 0.32	95.08 ± 5.87
LPS	0.64 ± 0.09 **	43.25 ± 2.83 **
pGCMV-IRES-EGFP + LPS	0.51 ± 0.08 **	41.89 ± 3.75 **
pGCMV-IRES-EGFP-14-3-3γ + LPS	1.90 ± 0.13 ^##^	76.41 ± 3.97 ^##^

The data are expressed as the mean ± SD from three separate experiments. * *p* < 0.05 and ** *p* < 0.01 *vs.* the control group; ^##^ *p* < 0.01 *vs.* the LPS group.

### 2.9. 14-3-3γ Overexpression Promoted the Secretion of β-Casein, Triglycerides and Lactose in LPS-Induced DCMECs

**Figure 8:**
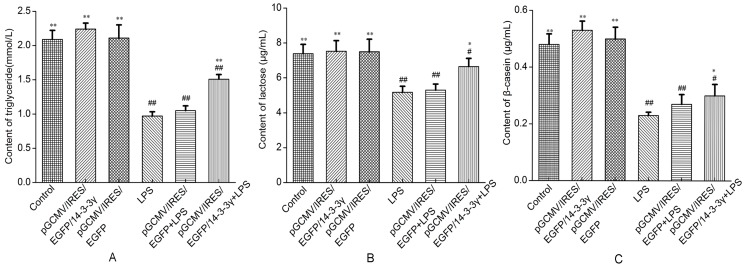
Effects of 14-3-3γ overexpression on the secretion of β-casein, triglycerides and lactose in LPS-induced DCMECs. DCMECs were pre-transfected with pGCMV-IRES-EGFP-14-3-3γ or pGCMV-IRES-EGFP for 24 h and then were treated with LPS (1 μg/mL). After 36 h, the content of β-casein, triglycerides and lactose were determined using an assay kit. (**A**) Triglyceride content; (**B**) lactose content; (**C**) β-casein content. Bars indicate the mean ± SD (*n* = 3). * *p* < 0.05 and ** *p* < 0.01 *vs.* LPS group; ^#^ *p* < 0.05 and ^##^ *p* < 0.01 *vs.* control group.

To evaluate whether the secretion of β-casein, triglycerides and lactose is associated with the inhibition of the inflammatory response by 14-3-3γ overexpression, the content of β-casein, triglycerides and lactose in the cell-free supernatants were determined using detection kits. Our results showed that LPS markedly reduced the secretion of triglycerides, β-casein and lactose compared to the control group (*p* < 0.01). However, 14-3-3γ overexpression promoted the secretion of β-casein, triglycerides and lactose in normal DCMECs compared to the control group, and it clearly increased the content of β-casein, triglycerides and lactose in LPS-induced DCMEC-free cell supernatants compared to the LPS group (*p* < 0.01 or *p* < 0.05) (Figure 8).

### 2.10. 14-3-3γ Overexpression Increased the Expression of Lactation-Associated Proteins in LPS-Induced DCMECs

**Figure 9:**
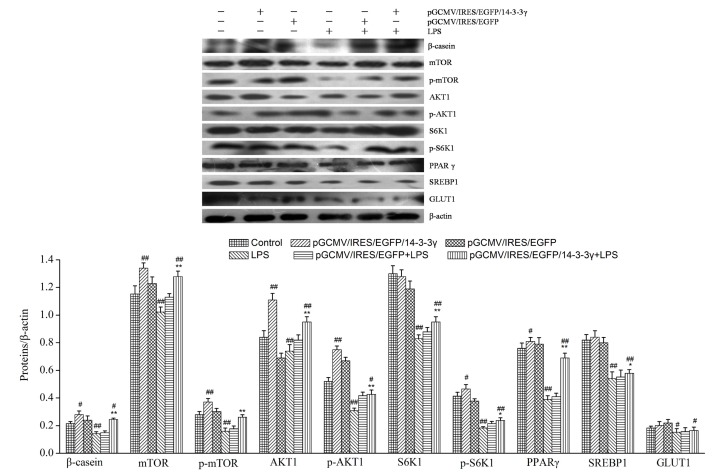
Effects of 14-3-3γ overexpression on lactation-associated proteins in LPS-induced DCMECs. DCMECs were pre-transfected with pGCMV-IRES-EGFP-14-3-3γ or pGCMV-IRES-EGFP. After 24 h, the cells were stimulated with LPS (1 μg/mL) for 24 h. Cytosolic proteins were analyzed by western blot for β-casein, mammalian target of rapamycin (mTOR), p-mTOR, ribosomal protein S6 kinase 1 (S6K1), p-S6K1, serine/threonine protein kinase Akt 1 (AKT1), p-AKT1, peroxisome proliferator activated receptor gamma (PPARγ), sterol regulatory element binding protein 1 (SREBP-1) and glucose transporter 1 (GLUT1). *β-Actin* was used as a control. Bars indicate the mean ± SD (*n* = 3). * *p* < 0.05 and ** *p* < 0.01 *vs.* LPS group; ^#^ *p* < 0.05 and ^##^ *p* < 0.01 *vs.* control group.

14-3-3γ can promote the expression of lactation-associated proteins or increase the protein activity of lactation signaling [18]. To evaluate whether lactation-associated signaling is up-regulated by 14-3-3γ overexpression in LPS-induced DCMECs, the protein expression of β-casein, mTOR, p-mTOR, S6K1, p-S6K1, AKT1, p-AKT1, PPARγ, SREBP1 and GLUT1 were measured by western blot. Our data demonstrated that treatment with 1 μg/mL LPS alone decreased the expression levels of β-casein, mTOR, S6K1, AKT1, PPARγ, SREBP1 and GLUT1compared to the control group (*p* < 0.01 or *p* < 0.05). Moreover, 14-3-3γ overexpression clearly increased the expression levels of β-casein, mTOR, S6K1, AKT1 and PPARγ in LPS-induced DCMECs compared to the LPS group (*p* < 0.01), and the expression levels of SREBP1 had increased (*p* < 0.05), but GLUT1 had little change. Meanwhile, 14-3-3γ overexpression increased the expression levels of β-casein, mTOR, AKT1 and PPARγ in normal DCMECs compared to the control group (*p* < 0.01 or *p* < 0.05), but SREBP1 and GLUT1 had little increase (Figure 9).

## 3. Discussion

Recently, extensive studies have been devoted to down-regulating LPS-induced inflammatory responses by regulating intracellular signaling molecules [24]. However, the role of 14-3-3γ as a signaling-regulated molecule remains relatively undeveloped. Only a few mechanisms have been reported thus far for 14-3-3γ [25]. Furthermore, to our best knowledge, the protective effect of 14-3-3γ on DCMECs induced by LPS has not been reported until now. In the present study, the anti-injury effects and mechanism of 14-3-3γ on LPS-induced DCMECs were measured. The data from this current study suggests that 14-3-3γ overexpression inhibits the production and expression of inflammatory cytokines by preventing NF-κB and MAPK activation in LPS-induced DCMECs; in addition, 14-3-3γ overexpression promotes lactation by increasing the phosphorylation levels of lactation-associated proteins. It has been suggested that 14-3-3γ might play a promising anti-inflammatory role by down-regulating inflammatory mediators and up-regulating the mTOR pathway, which may play a potential role in preventing the inflammation induced by SARA.

It has previously been reported that the secretion of TNF-α, IL-6 and IL-1β instantly increased when LPS stimulated the mammary gland, which initiated acute phase protein synthesis and depressed the synthesis of milk composition [26,27]. To verify whether inflammatory factors could be produced in LPS-induced DCMECs, we treated DCMECs with LPS. The data in our study showed that the expression of TNF-α, IL-6 and IL-1β mRNA was significantly increased (Figure 4). Meanwhile, the concentrations of IL-6 and TNF-α in the DCMEC culture supernatant were dramatically increased after treatment with LPS (Figure 5). Additionally, LPS stimulation leads to the generous expression of inducible nitric oxide synthase (iNOS) [11], and the production of NO increases in the mammary glands [28], whereas enzymes linked to NO metabolism have recently been shown to considerably affect the milk composition of inflamed mammary glands [29]. In the present study, we have demonstrated that the expression of iNOS mRNA was significantly increased compared to DCMECs without LPS treatment. These observations are consistent with previous studies.

It has been reported that 14-3-3γ is up-regulated following cardiac damage, and that it most likely acts as a stress factor to protect against cardiac damage [30]. In our study, the changes in 14-3-3γ mRNA and protein levels after LPS stimulation in DCMECs were measured, and the results showed that the relative expression level of 14-3-3γ increased significantly after LPS treatment, which is consistent with previous studies [31]; afterwards, 14-3-3γ expression decreased steadily (Figure 2). The mechanism of LPS-induced the change of 14-3-3γ expression in DCMECs requires further study. This variety in 14-3-3γ expression level implies that 14-3-3γ may take part in the organism against injury induced by LPS.

We then aimed to determine whether a high level of 14-3-3γ protein could inhibit the production of inflammatory mediators. We successfully constructed the pGCMV-IRES-EGFP-14-3-3γ plasmid and used it to transfect primary cultured DCMECs 24 h before LPS treatment. Because xenobiotics could induce inflammatory cytokines, we set pGCMV-IRES-EGFP-14-3-3γ as the control group to clarify whether the production of inflammation was due to LPS. We determined that 14-3-3γ overexpression attenuated the production of IL-6 and TNF-α in the cell culture supernatants and inhibited expression of TNF-α, IL-6, iNOS and IL-1β in LPS-induced DCMECs (Figure 4 and Figure 5). These results indicate that 14-3-3γ plays an anti-inflammatory role in LPS-induced DCMECs.

To further explore the mechanism of the inhibitory effect of 14-3-3γ overexpression on cytokine production, we evaluated the MAPK and NF-κB signal transduction pathways that were activated by LPS. It is known that the activation of MAPK is involved in the regulation of the LPS-stimulated release of immune-related cytotoxic factors and proinflammatory cytokines [32]. In our study, upon transfection of DCMECs with the pGCMV-IRES-EGFP-14-3-3γ plasmid, we found that LPS-induced DCMECs had significantly increased phosphorylation of ERK1/2, p38 MAPK and JNK; however, 14-3-3γ overexpression markedly inhibited the phosphorylation of ERK1/2 and p38 MAPK, but not of JNK. The results showed that 14-3-3γ overexpression inactivated MAPK signaling pathways by down-regulating ERK, and p38 MAPK, thereby inhibiting cytokine production in LPS-induced DCMECs (Figure 6). NF-κB is an important signaling molecule that plays a key role in the development of inflammatory diseases [33,34]. When cells are activated by LPS, IκB is phosphorylated by IκB kinase and degraded, resulting in the NF-κB p65 subunit dissociating from its inhibitory protein, IκBα, and translocating from the cytoplasm to the nucleus, where they may generate a variety of proinflammatory cytokines [35]. Our study showed that 14-3-3γ overexpression suppresses the phosphorylation of IκBα in LPS-induced DCMECs. Additionally, NF-κB translocation was detected in the nuclei of LPS-induced DCMECs, but it was inhibited when 14-3-3γ was overexpressed. 14-3-3γ overexpression inactivated the NF-κB signaling pathways by down-regulating IκB phosphorylation and inhibiting NF-κB translocation in LPS-induced DCMECs (Figure 7). Based on the above data, our results suggest that 14-3-3γ overexpression suppressed LPS-induced pro-inflammatory cytokine production by preventing NF-κB and MAPK activation in LPS-stimulated DCMECs.

It has long been assumed that LPS is responsible for the activation of systemic inflammation and other important metabolic disturbances of the host and causes depression of milk production [36]. It is well known that the number and activity of mammary epithelial cells are closely related to lactation, and an increased number of mammary epithelial cells and enhanced cell viability will contribute to lactation [37]. LPS is one of the most potent microbial inducers of inflammation, inducing the activation of executioner caspases and other signaling cascades that ultimately lead to apoptosis of the cells [38]. Our results showed that 14-3-3γ overexpression may inhibit the inflammatory cytokines induced by LPS in DCMECs. However, it remains unclear whether 14-3-3γ overexpression is responsible for variations in the activity and number of cells in LPS-induced DCMECs. In addition, the major milk protein β-casein, lactose and milk fat are the primary indicators for evaluating lactation capacity and milk quality [39,40]. Therefore, the cell proliferation and secretion of β-casein, lactose and triglycerides were measured. In these studies, our results showed that 14-3-3γ overexpression had a positive effect on cell proliferation and viability of LPS-induced DCMECs (Table 1), which may be relevant to inhibition of cytokine production; similar results have been reported [24]. Furthermore, our results also demonstrated that LPS markedly inhibited the secretion of triglycerides, lactose and β-casein in DCMECs; this result was consistent with previous studies indicating that LPS challenge induced a significant transient decrease in lactose concentration and casein secretion [41]. However, 14-3-3γ overexpression clearly increased the concentration of β-casein, triglycerides and lactose and the synthesis of β-casein in LPS-induced DCMECs (Figure 8). These data imply that 14-3-3γ has a protective effect on LPS-induced injury.

There are many transcription factors related to lactation. mTOR plays an important role in the growth of DCMECs and milk protein synthesis [42,43]. S6K1 is a down-stream target of mTOR; it plays an important role in cell proliferation and cell cycle progression [44]. AKT1 is an important regulatory factor in cell signal transduction pathways, and the PI3K/Akt pathway is essential for the synthesis of milk components such as lipids and lactose [45]. PPARγ, a ligand-dependent nuclear transcription factor, is a key regulatory factor in the synthesis of milk fat [46,47]. SREBP1 is a member of the basic helix-loop-helix transcription factor family, capable of activating the transcription of genes for the synthesis of fatty acids [48]. GLUT1 is the major glucose transporter in the basal membrane [49]. In our study, we found that a reduction in the β-casein, mTOR, S6K1, AKT1, PPARγ, SREBP1 and GLUT1 levels in the DCMECs was induced by LPS. In contrast, when 14-3-3γ was overexpressed, the expression levels of β-casein, mTOR, S6K1, AKT1, PPARγ and SREBP1 increased. And 14-3-3γ overexpression also increased the expression of β-casein, mTOR, AKT1, PPARγ, SREBP1 and GLUT1 in the DCMECs (Figure 9). A previous study showed that 14-3-3γ could effectively increase the expression of mTOR and AKT1. We speculate that 14-3-3γ overexpression principally up-regulates the mTOR/AKT1 signaling pathway to increase lactation.

**Figure 10:**
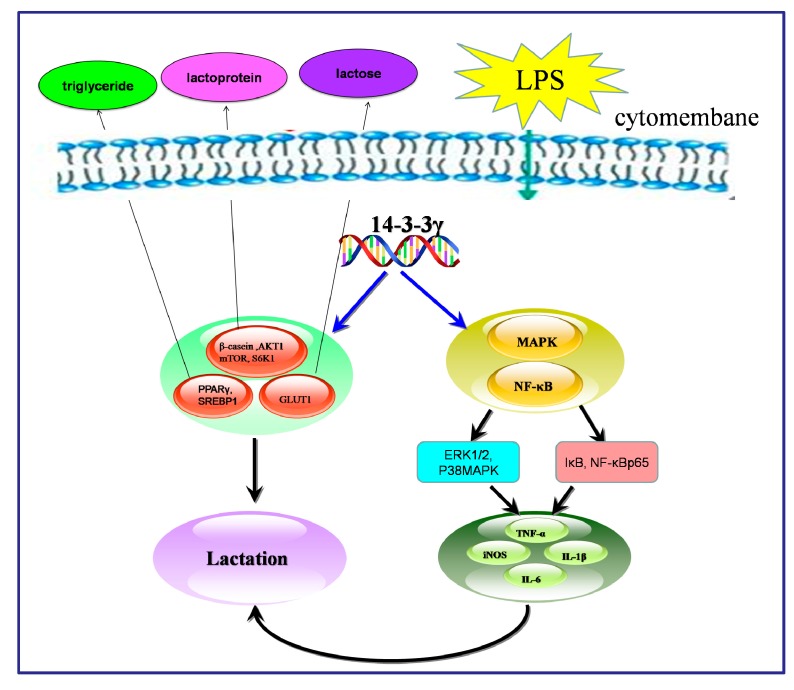
Diagram summarizing our findings. 14-3-3γ may have a beneficial effect in depressing LPS-induced inflammatory response in DCMECs through inhibiting NF-κB and MAPKs and up-regulating mTOR signaling pathway. Green arrows indicated that LPS get into the cell membrane; Blue arrows indicated that 14-3-3γ regulated genes expression; Black arrows indicated that change of genes expression had effects.

In summary, our findings suggest that 14-3-3γ may have a beneficial effect in preventing LPS-induced inflammatory response in DCMECs. The mechanisms by which 14-3-3γ overexpression can exert its anti-inflammatory effects correlate with the inhibition of the expression of TNF-α, IL-6, IL-1β and iNOS via the inactivation of the NF-κB and MAPK signaling pathways. Meanwhile, 14-3-3γ overexpression can promote lactation by up-regulating mTOR, S6K1, AKT1, PPARγ and SREBP1 expression in LPS-induced DCMECs (Figure 10). Thus, we conclude that the high level of 14-3-3γ may play important protective roles in LPS-induced DCMEC damage and milk quality depression by inhibiting NF-κB and MAPKs and up-regulating the mTOR signaling pathway.

The study has reported that nutrients can inhibit the expression of inflammatory cytokines [50]. Furthermore, previous studies showed that lysine, methionine and prolactin could effectively increase expression of the 14-3-3γ gene promoter, and inhibit methylation of promoters [18]. Combined with previous reports, we hypothesize that adding nutrients to the dairy cow diet during lactation to regulate the expression of 14-3-3γ protein, and thereby acting against SARA on the protein level, could offer an opportunity for therapeutic intervention.

## 4. Experimental Section

### 4.1. Chemicals and Reagents

Lipopolysaccharide (LPS, *Escherichia coli* 0111:B4) was obtained from Sigma Chemical Co. (St. Louis, MO, USA). Fetal bovine serum (FBS) and Dulbecco’s modified Eagle medium/F12 (DMEM/F12) base were obtained from GIBCO BRL (Life Technologies, Carlsbad, CA, USA). ELISA kits for TNF-α and IL-6 were purchased from Beijing Keyanmei Technology Co., Ltd. (Beijing, China). The lactose/d-galactose (Rapid) assay kit was obtained from Megazyme (Ireland, UK). The ELISA kit for β-casein was obtained from New England Biolabs Inc. (Beverly, MA, USA). Antibodies to 14-3-3γ, JNK2, phospho-JNK2, phospho-p38 MAPK, p38 MAPK, phospho-ERK1/2, ERK1/2, phospho-IκB-α, IκB-α, NF-κB p65, Proliferating cell nuclear antigen (PCNA) and β-actin were purchased from Santa Cruz Biotechnology, Inc. (Santa Cruz, CA, USA). Antibodies to β-casein were purchased from Abbiotec (San Diego, CA, USA). The pGCMV-IRES-EGFP-14-3-3γ expression vector was provided by the Dairy Science Key Laboratory of the Education Ministry of Northeast Agricultural University.

### 4.2. Identification and Culture of DCMECs

Dairy cow mammary epithelial cells obtained from the bovine mamary gland parenchymal tissues were individually isolated and purified from mid-lactation Holstein dairy cows according to previous published reports [51]. All animals received humane care as outlined in the Guide for the Care and Use of Experimental Animals of the National Institutes of Health. The procedure was approved by the Ethics Committee of Northeast Agricultural University. All surgeries were conducted with an effort to minimize suffering to animals.

The cells were purified by digesting with 0.25% trypsin 3 passages, and the purified cells were cultured in basic culture medium (DMEM/F12 base with 10% fetal bovine serum, 100 U/mL penicillin and 100 U/mL streptomycin). For experimental assays, the cells were maintained in a culture bottle with medium at 37 °C, 5% CO_2_. DCMECs were identified by cytokeratin-18 (CK18) according to the previously published method of Li *et al.* [52]. DCMECs were seeded in a laser confocal petri dish and cultured until to 30%–50% confluency, then the cells were washed three times with PBS and fixed in 4% (*w*/*v*) pre-cooled formaldehyde for 15 min. After 15 min, the formaldehyde was poured out and the cells were washed three times with PBS for 5 min per wash. The fixed DCMECs were incubated in blocking buffer (Tris-buffered saline with 5% BSA and 0.1% TritonX-100, TBS/T) for 1 h at 37 °C, then incubated with anti-CK18 primary antibody at a 1:50 dilution for 1 h at 37 °C. After washing three times in TBS/T, the specimens were incubated in the dark with fluorescein isothiocyanate (FITC)-conjugated second antibodies at a 1:100 dilution for 1 h at 37 °C, washed twice in TBS/T and incubated with propidium iodide (PI) or 10 min at room temperature. Finally, after washing three times in TBS/T, the dish was visualized using a TCS-SP2 AOBS laser scanning confocal microscope (Leica Microsystems, Heidelberg, Germany).

### 4.3. Effect of LPS on Expression of 14-3-3γ mRNA

DCMECs were reseeded at a density of 3 × 10^5^ cells/cm^2^ in 6-well tissue culture plates and incubated with basic culture medium (DMEM/F12 base with 10% fetal bovine serum, 100 U/mL penicillin and 100 U/mL streptomycin) for 24 h. The cells were treated with 1 μg/mL LPS from *Escherichia coli* 0111:B4 for different durations (0, 3, 6, 12 and 24 h) to detect the level of change in 14-3-3γ mRNA. The expression of the 14-3-3γ gene was measured by RT-PCR.

### 4.4. Transfection of pGCMV-IRES-EGFP-14-3-3γ (Expression Vectors)

DCMECs were cultured in 6-well plates at a density of 3 × 10^5^ cells/cm^2^. When cells reached 80%–90% confluence, they were washed twice with D-Hanks and incubated with 1.5 mL basic culture medium but without penicillin and streptomycin. The plasmid pGCMV-IRES-EGFP-14-3-3γ was transfected into cells using Lipofectamine™ 2000 (LF2000, Invitrogen, Camarillo, CA, USA) according to the manufacturer’s instructions. A volume containing 4 μg plasmid and 10 μL Lipofectamine™ 2000 was diluted into 250 μL medium without serum. Then, the above two were combined and incubated at room temperature for 20 min. The complexes were added onto the cultured cells. Four hours later, the medium was discarded and fresh culture medium was added. After 24 h transfection, the expression of 14-3-3γ proteins was measured by western blot.

### 4.5. Quantification of Tumor Necrosis Factor-α (TNF-α) and Interleukin-6 (IL-6)

DCMECs were pre-transfected with pGCMV-IRES-EGFP-14-3-3γ or pGCMV-IRES-EGFP for 24 h. The transfected cells were treated with or without 1 μg/mL LPS for 6 h. Then, the cell-free supernatants were collected for the TNF-α and IL-6 cytokine concentration assays using bovine enzyme-linked immunosorbence assay (ELISA) kits, according to the manufacturer’s instructions. The optical density (OD) of the microplate was read at 450 nm.

### 4.6. RNA Extraction and Quantitative Real-Time PCR

Total RNA was isolated using Trizol reagent according to the method previously published by Lu *et al.* [53,54]. The total RNA was reverse transcribed into cDNA using Primescript reverse transcriptase (TaKaRa, Dalian, China) according to the manufacturer’s protocol. The mRNA levels of various genes were quantified using SYBR premix Ex Taq™ (Takara, Tokyo, Japan), and analysis was performed using an ABI PRISM 7300 RT-PCR System (Applied Biosystems, Foster City, CA, USA). The primers used for RT-PCR analysis are presented in Table 2. RT-PCR conditions were: 95 °C for 30 s and then 40 cycles at 95 °C for 5 s and 60 °C for 31 s, according to the manufacturer’s instructions. All the target cDNAs were analyzed in triplicate. Data was analyzed using the 2^−ΔΔ*C*t^ method.

**Table 2:** Sequence of primers used in current investigation in qRT-PCR.

Gene Name	Primer Sequence (5′–3′)	Product Size (bp)	GenBank
*14-3-3γ*	Forward: GCCGTATGTCAGGATGT	171	BC153255.1
	Reverse: GCCAGGTAGCGGTAAT		
*TNF-α*	Forward: GCCGTATGTCAGGATGT	140	AC000180.1
	Reverse: GCCAGGTAGCGGTAAT		
*IL-6*	Forward: TGAGGGAAATCAGGAAAATGT	269	AC000161.1
	Reverse: CAGTGTTTGTGGCTGGAGTG		
*IL-1β*	Forward: AGGTGGTGTCGGTCATCGT	190	NC019460.1
	Reverse: GCTCTCTGTCCTGGAGTTTGC		
*iNOS*	Forward: CAGCCCCCGTCCAGTCCAGTGA	186	AC000176.1
	Reverse: GACTCATTCCCGTGCTTGCCCG		
*β-actin*	Forward: CCGCAAGGACCTCTACGC	206	AC000182.1
	Reverse: CATGCCAATCTCATCTCGTTTT		

### 4.7. Assessment of Viability and Proliferation of DCMECs

DCMECs were pre-transfected with pGCMV-IRES-EGFP-14-3-3γ or pGCMV-IRES-EGFP for 24 h. Transfected cells were treated with or without 1 μg/mL LPS for 6 h. Cell viability and proliferation were measured using a CASY-TT Analyzer System (Schärfe System GmbH, Reutlingen, Germany) according to the manufacturer’s instructions. DCMECs were digested with trypsin and then diluted (1:100) with CASY electrolyte solution prior to examination. Three 100 μL aliquots were analyzed for each sample [55].

### 4.8. Determination of β-Casein, Triglycerides and Lactose

DCMECs were pre-transfected with pGCMV-IRES-EGFP-14-3-3γ or pGCMV-IRES-EGFP for 24 h. Transfected cells were treated with or without 1 μg/mL LPS for 36 h. Then, cell-free supernatants were collected to measure the concentrations of β-casein, triglyceride and lactose using the ELISA kits for β-casein, triglycerides quantitative assay kit and lactose/d-galactose (Rapid) assay kit (Megazyme, Bray Business Park, Bray, Ireland), respectively, according to the manufacturer’s instructions.

### 4.9. Western Blot Analysis

DCMECs were pre-transfected with pGCMV-IRES-EGFP-14-3-3γ or pGCMV-IRES-EGFP. After 24 h, the cells were stimulated with or without 1 μg/mL LPS for 1 h. The cells were collected and washed twice with cold PBS. The cells were lysed in lysis buffer (Cell Signaling Technology Inc., Danvers, MA, USA). Nuclear protein was isolated from the DCMECs using the method reported by Li *et al.* [56]. Protein concentrations were measured using the BCA method, and 30 µg of protein was separated on a 10% SDS-PAGE gel and transferred onto nitrocellulose membranes with glycine transfer buffer (192 mM glycine, 25 mM Tris-HCl (pH 8.8), 20% methanol). The membranes were blocked in blocking buffer (5% nonfat dry milk) for 1.5 h at room temperature. The membranes were incubated overnight with specific primary antibodies at 4 °C. The membranes were washed three times with TBS/T and then incubated for 60 min with horseradish peroxidase (HRP)-conjugated antibodies at 37 °C. The immunoactive proteins were measured using Super ECL plus (ApplyGEN, Beijing, China). Analysis of the western blot results was subsequently performed using the Tanon 1600R automatic digital gel image analysis system (Tanon Science & Technology Co., Ltd., Shanghai, China)

### 4.10. Statistical Analysis

Quantitative data are expressed as the mean ± standard deviation (SD) for each group from three independent experiments. Differences between the mean values of normally distributed data were analyzed using one-way ANOVA with SPSS 17.0 statistical software (SPSS, Inc., Chicago, IL, USA). Statistical significance was declared at *p* < 0.05 or *p* < 0.01.
